# Aspiration of superabsorbent polymer beads resulting in focal lung damage: a case report

**DOI:** 10.1186/s12887-020-02168-9

**Published:** 2020-05-29

**Authors:** Nasser Alharbi, Maryam Dabbour

**Affiliations:** 1 Department of Pediatrics, College of Medicine, King Saud University and King Saud University Medical City, Riyadh, Saudi Arabia; 2 Pediatric Pulmonology, Prince Sultan Military Medical City, Riyadh, Saudi Arabia

**Keywords:** Orbeez, Water-absorbing bead, Bronchiectasis, Foreign body aspiration

## Abstract

**Background:**

Concerns have recently been raised about reported incidents of intestinal obstruction following ingestion of Superabsorbent polymer (SAP) beads. Texas Poison Centers reported 110 cases of superabsorbent polymer bead ingestions between 2011 and 2016 (Pediatr Emerg Care 35:426-7, 2019). Furthermore, cases of related auditory complications following the placement of SAP beads into the external auditory canal have also been reported. Here, we report the first case of significant airway damage secondary to the ingestion of a SAP bead (Orbeez), which was aspirated and then overlooked. Further, we hypothesized that the capability of the bead to expand in size once exposed to water from the respiratory mucous may contribute to airway damage.

**Case presentation:**

A 3-year-old boy presented to our hospital with persistent cough and recurrent hospitalizations to the general ward and intensive care unit. The boy was diagnosed with focal lung bronchiectasis in the left lower lobe, which occurred after the patient aspirated an Orbeez bead before a year. The bead was removed using flexible bronchoscopy and a retrieval basket.

**Conclusion:**

Orbeez beads are commonly ingested by young children resulting in gastrointestinal obstruction. The beads can easily be aspirated by children and overlooked by their caregivers and physicians for long periods of time due to their small size. The bead can cause significant airway damage after multiplying in size when coming into contact with respiratory mucus which consists of 95% water.

## Background

Foreign body aspiration is relatively common public health problem. Accounting for 7% of accidental deaths in children under 4 years of age [1, 2]. Despite that 25% of the inhaled foreign body are non-organic material, they represent the major indication for surgical interventions [3, 4]. The symptoms resulting from FB aspiration are related to size, shape, and nature of the foreign body [5]. Whereas organic foreign bodies cause airway inflammation, an inorganic foreign body may go undetected for a long time without causing any remarkable symptom [2–4].

The longer the FB remains in the bronchial tree the greater the chances are of it becoming dislodged from the bronchus and impacting the larynx, with risk being higher in inorganic FBs [6].

Superabsorbent polymer (SAP) bead capable of increasing its original size up to 200 times once placed in water [7]. It has the appearance of candy, and thus, is commonly ingested by children [8, 9]. From 2011 to 2016, the Texas Poison Center Network (TPCN) reported 110 incidents of SAP bead ingestion. Half of these incidents occurred in school-aged children, and around 30% of these incidents happened at school [8]. Serious intestinal obstruction following the ingestion of these beads has been reported [7, 10]. A brand of SAP beads called Water Balz was recalled by the U.S. Consumer Product Safety Commission in 2012 due to its potential hazard on the gastrointestinal system [8]. Cases of SAP bead insertion into the auditory canal have been reported as well, in one incident, a young girl experienced significant external auditory canal and cochlear erosion with profound sensorineural hearing loss as a result of an overlooked SAP in the right ear [11, 12].

In this study, we report the first case of significant focal lung damage as a result of an Orbeez bead ingestion which expanded in size after coming into contact with lower airway secretions. Thus, this study necessitates spreading public awareness of the potential danger of SAP beads.

## Case presentation

**Fig. 1:**
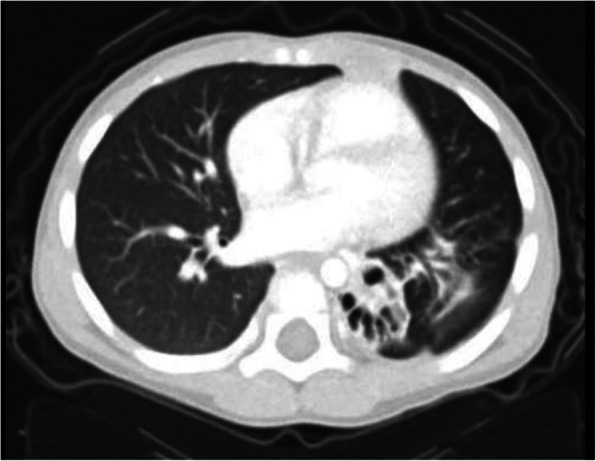
Chest CT showed a significant damage of the affected segment

A 3-year-old boy was referred to the King Faisal Specialist Hospital and Research Center (KFSH&RC) from the regional hospital in Al-Ahsa region in the eastern province of Saudi Arabia. The patients experienced recurrent chest infections associated with a persistent wet cough which resulted in four hospitalizations over the last year. The child was born uneventfully after stable pregnancy and had no remarkable background history in the first 2 years of his life apart from infrequent mild upper respiratory tract infections without symptoms, suggestive of a lower respiratory tract infection. Shortly after his second birthday, the boy had the first onset of lower respiratory tract infections and presented to the local hospital with fever, tachypnea, and respiratory distress, significant enough to require ICU admission. He responded partially to the initial regimen with oxygen therapy, systemic antibiotics, and systemic steroid and nebulized bronchodilators during the hospitalization. His cough persisted after he was discharged from the hospital and he soon suffered from additional attacks during which his symptoms worsened. His symptoms included respiratory distress, an aggravated cough, and hypoxia, and required frequent emergency visits and a total of 4 hospitalizations over the course of 1 year. The patient’s parents are *non-consanguineous,* and there was no history of respiratory infections or symptoms in the family. Upon examination of the patient’s immune system, test results showed normal immunoglobulin levels and normal lymphocyte markers. A computed tomography (CT) scan of the chest (Fig. 1) indicated multiple cystic changes within the posterior segment of left lower lobe, which most likely represented bronchiectasis changes. FB ingestion was suspected after radiology; however, congenital lung malformation could not be ruled out. At this point, the clinical and radiological presentation were suggestive of a foreign body had been ingested by the child and was overlooked. Consequently, the child was referred to our facility where we performed flexible bronchoscopy after the boy had been ill for 1 year.

**Fig. 2:**
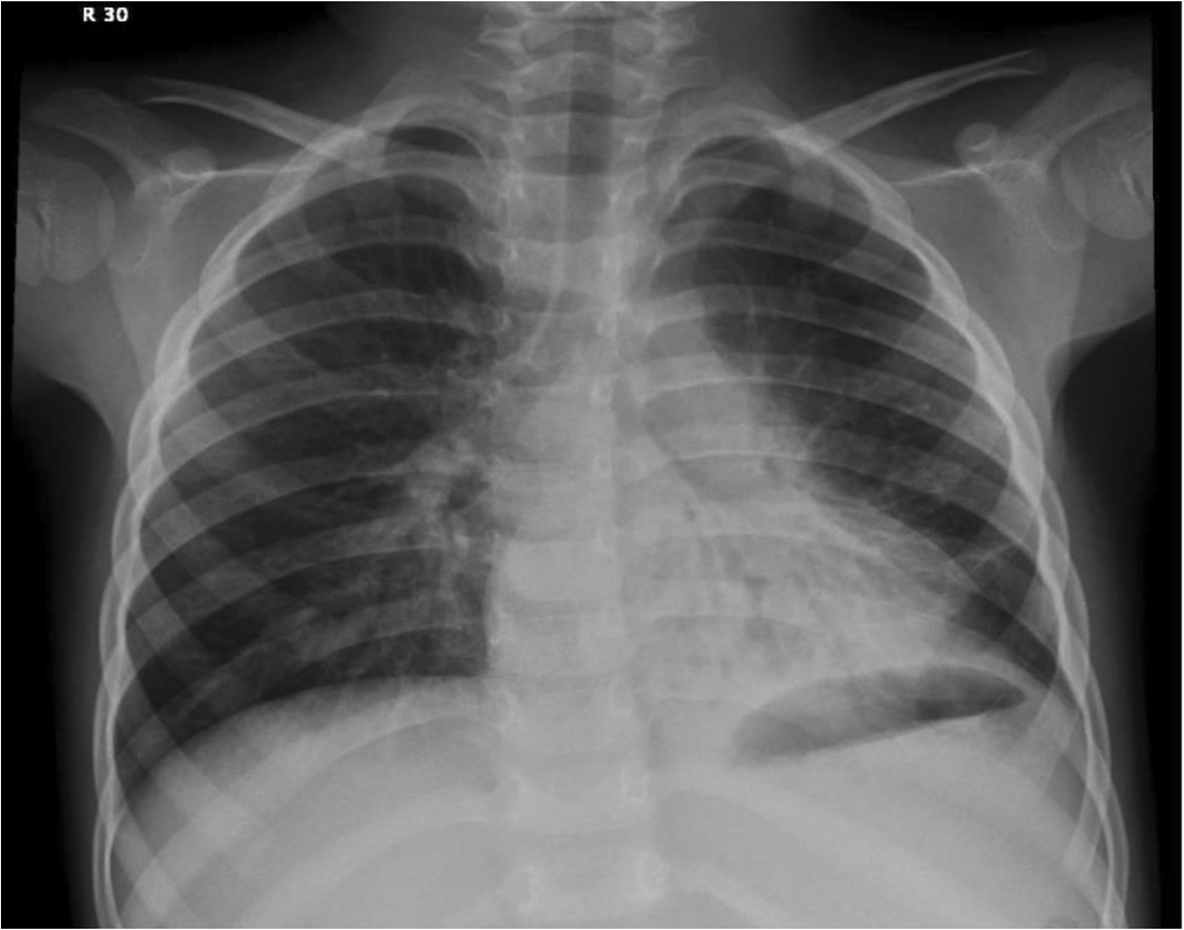
Chest X-ray before the bronchoscopy showing a significant lower lobe consolidation with Bronchiectasis in in the same lobe

**Fig. 3:**
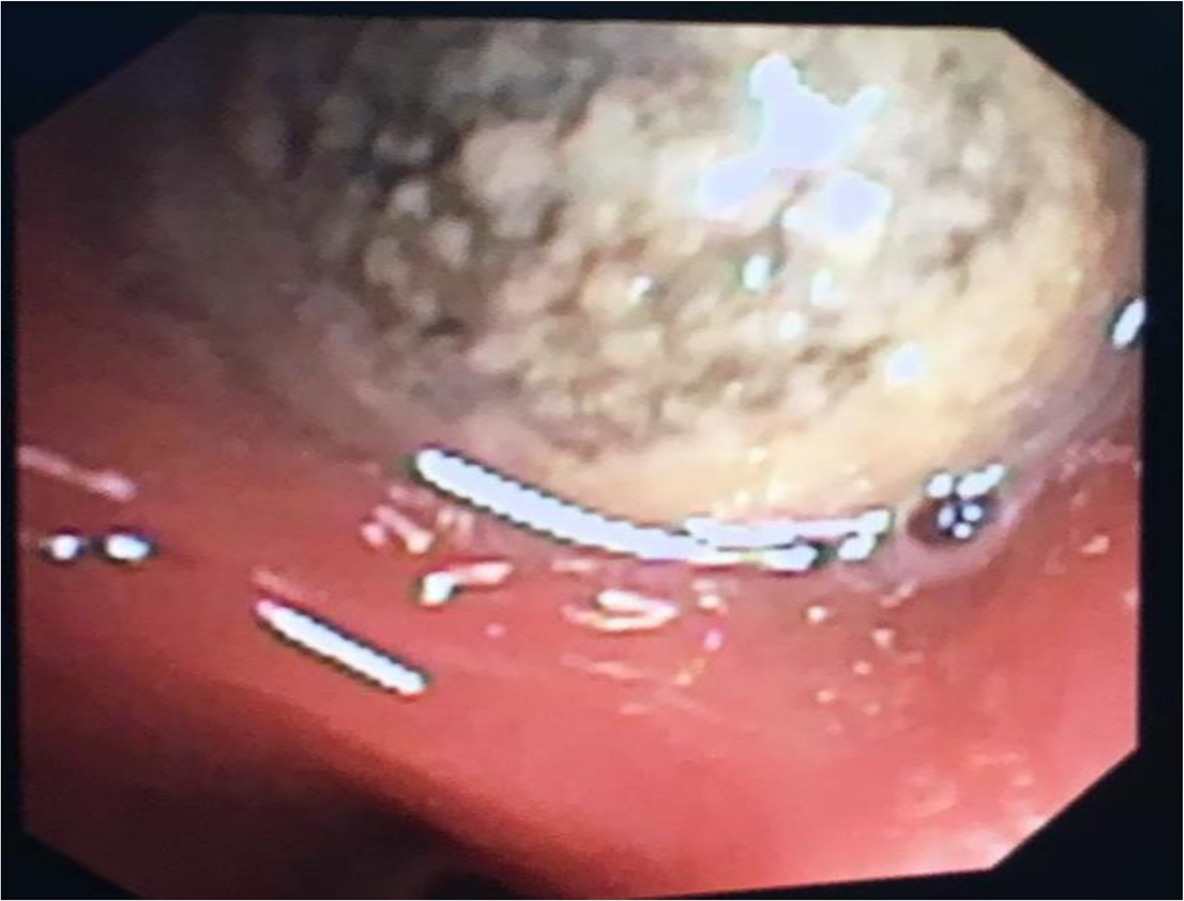
A granulation tissue covered the object blocking the airway completely, causing pressure over the adjacent wall and squeezing the rest of the airways

Clinical examination performed at our facility demonstrated a normal oxygen saturation on room air. There was a decrease in air entry in the left lower zone with no adventitious sounds. Initial chest x-ray (Fig. 2) showed a significant left lower lobe consolidation with Bronchiectasis. Flexible bronchoscopy was performed under general anesthesia, and the patient was intubated by an endotracheal tube in the operating room. A sphere-shaped foreign body (Fig. 3) was identified in the left lower lobe posterior segment. The patient’s parents were certain that the foreign body represented an Orbeez ball.

**Fig. 4:**
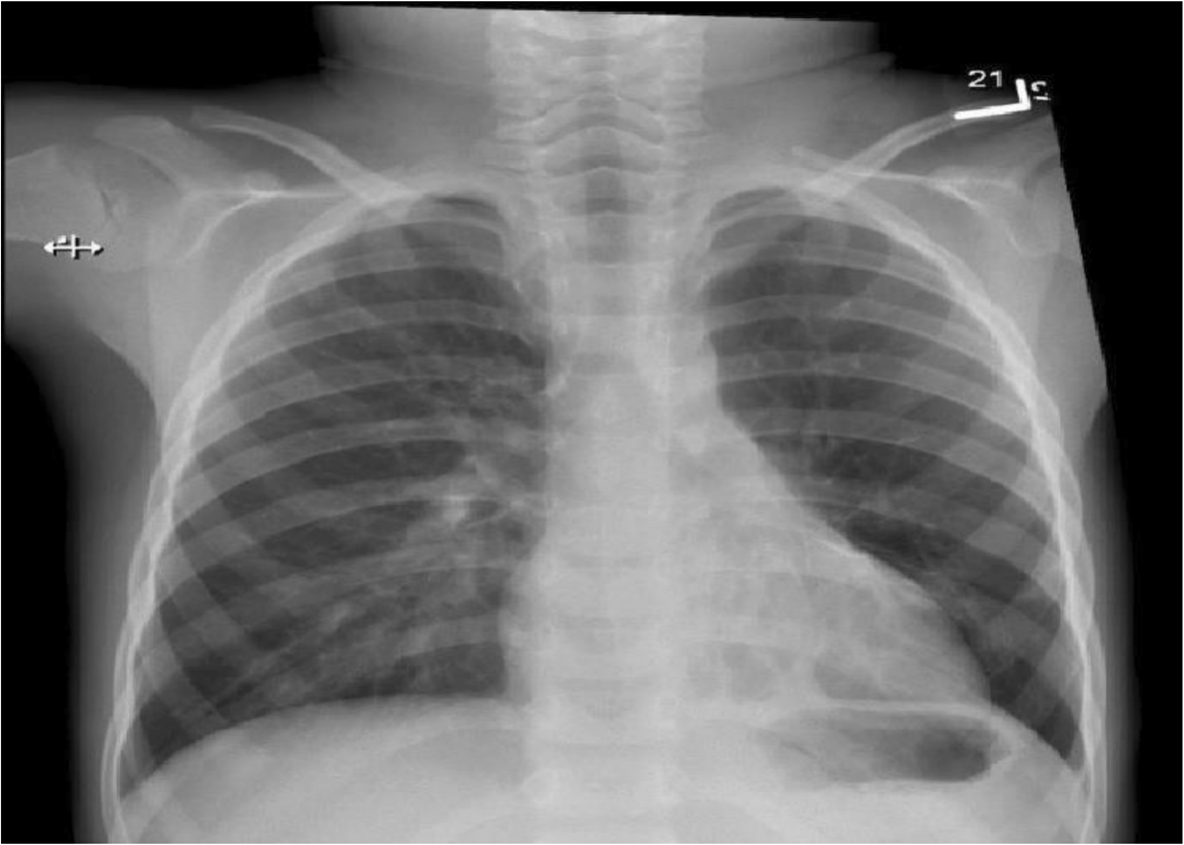
Chest X-Ray 6 months after Bronchoscopy showing a significant varicose bronchiectasis in the left lower lobe with interval improvement of the consolidation in the same lobe

An attempt was made to remove the foreign body using a retrieval basket and a flexible scope. Unfortunately, the foreign body was squashed and fragmented into smaller pieces, which required the removal of each piece using the retrieval basket. Eventually, all fragments were removed successfully. We observed that granulation tissue was causing a significant obstruction of the left lower lobe posterior segment airway, to the extent that we could not pass the scope through it. After reconstructing the pieces of the foreign body, the patient’s parent indicated that it appeared much larger than its original size. The fragmented pieces were reviewed by a pathologist who confirmed the inorganic nature of the foreign body. At an evaluation after 6 months following the removal of the foreign body the child’s symptoms were minimal, with no additional hospitalizations recorded. His chest X-ray demonstrated a persistent bronchiectasis in left lower lobe with interval improvement in the previously noted consolidation (Fig. 4).

## Discussion and conclusion

SAP beads are small round objects made of inorganic material. They are an inexpensive and widely available children’s toy with the appearance of candy [7].

Concerns have been raised after various reports of intestinal obstruction following ingestion of these beads [7, 10].

Our study described a case in which the immediate symptoms were unremarkable. Nevertheless, after coming into contact with respiratory mucus which consists of 95% water, the beads increased in size, causing damage to the lungs [13]. If swallowed, the beads have the potential to remain undetected for long periods of time, our study described a case in which the immediate symptoms were unremarkable. In this case report, the patient developed significant focal bronchiectasis as a result of SAP bead ingestion [13]. Using flexible bronchoscopy and a retrieval basket to retrieve the FB can be a reasonable first line intervention.

Age restriction and parent observation are not an effective way to prevent these SAP ingestion from occurring, as nearly half of the reported incidents occurred in school-aged children, with 30% occurring while the children were at school [8].

Thus far, studies have reported that toys such as SAP beads to cause harm to the intestines, the external auditory canal, and as in our case, the respiratory system. We suspect that if stuck in the larynx and enlarged after absorbing water, SAP beads may result in a catastrophic sequelae.

For the above mentioned reasons, we propose recalling all brands of SAP beads from markets. In the meantime, we suggest that every incidence of complications following the ingestion of SAP beads be reported.

## Data Availability

Data sharing is not applicable to this report as no data sets were generated or analyzed.

## Abbreviations

FB: foreign body
KFSH&RC: King Faisal specialist hospital and research center
ICU: Intensive Care Unit
CT: computed tomography
SAP: Superabsorbent Polymer
